# Quartz resonator assembling with TSV interposer using polymer sealing or metal bonding

**DOI:** 10.1186/1556-276X-9-541

**Published:** 2014-10-01

**Authors:** Jian-Yu Shih, Yen-Chi Chen, Chih-Hung Chiu, Chung-Lun Lo, Chi-Chung Chang, Kuan-Neng Chen

**Affiliations:** 1 Department of Electronics Engineering, National Chiao Tung University, Hsinchu 300, Taiwan; 2 TXC Corporation, Taoyuan 324, Taiwan

**Keywords:** Three-dimensional (3D) integration, Through-silicon via (TSV), Wafer thinning, Sealing bonding

## Abstract

This paper presents one wafer level packaging approach of quartz resonator based on through-silicon via (TSV) interposer with metal or polymer bonding sealing of frequency components. The proposed silicon-based package of quartz resonator adopts several three-dimensional (3D) core technologies, such as Cu TSVs, sealing bonding, and wafer thinning. It is different from conventional quartz resonator using ceramic-based package. With evaluation of mechanical structure design and package performances, this quartz resonator with advanced silicon-based package shows great manufacturability and excellent performance to replace traditional metal lid with ceramic-based interposer fabrication approach.

## Background

Conventional quartz resonator components are packaged in metal lid with ceramic-based interposer enclosures. In order to keep frequency components from moisture or other contamination and mechanical attack, quartz resonator requires specialized packaging with the protective cap over the quartz resonator blank [1,2]. With these technologies of metal lid and ceramic substrate, current quartz resonator can be fabricated in a size of 1.6 × 1.2 mm. However, challenges and issues of current manufacturing of ceramic-based quartz resonator package, including high-cost packaging material of metal lid, one chip sealing bonding, and scaling limit of ceramic substrate, make the shrinkage of quartz resonator packages difficult. For this reason, to develop a new packaging method with quartz resonator is necessary to solve the abovementioned difficulties and achieve a high performance, small form factor, and low manufacturing cost [3,4].

In recent years, with the development of wireless communication, the small form factor, low cost, and high-performance microelectronic products become significant due to the need of market. Multi-chips integration is the trend of the present semiconductor and package industries due to advantages mentioned above. Among the multi-chips integration techniques, three-dimensional (3D) integration is one of the most promising approaches in microelectronic integration and package techniques [5-11]. 3D integration technologies achieve heterogeneous integration and offer a smaller area solution for electronic products by the Cu through-silicon via (TSV) interconnection and vertical stacking. Several 3D integration schemes have been proposed in worldwide institutes to establish 3D integration platforms [12-17]. The 3D scheme combined with sealing bonding techniques by using sealing wafer level packaging of micro electro mechanical system (MEMS) component is successfully achieved, such as SAW RF Filter, silicon MEMS oscillators, and image sensor devices [18-22].

**Figure 1:**
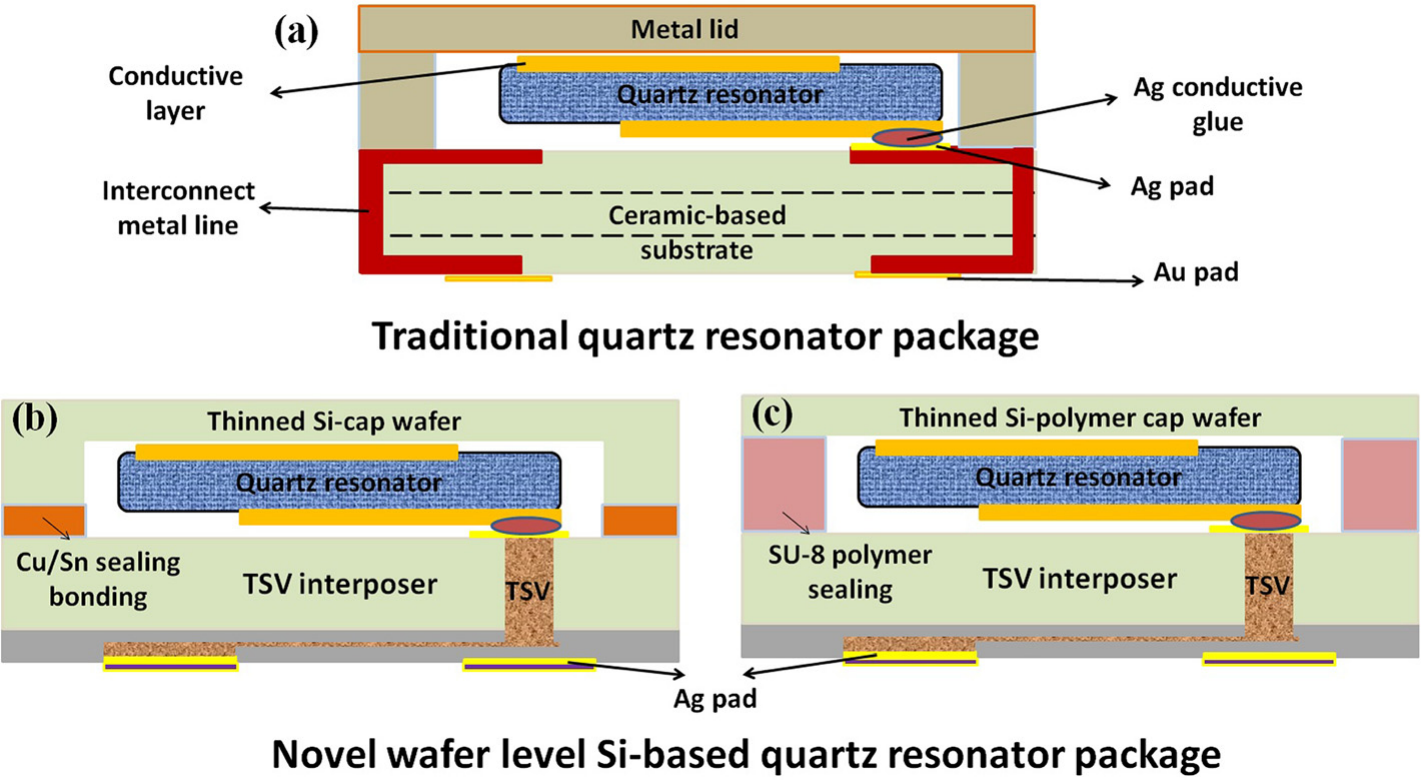
Schematics of (a) traditional quartz resonator and (b,c) novel silicon-based quartz resonator.

In this research, an innovated assembling design of quartz resonator package using TSV interposer is proposed not only to replace ceramic substrate but also to solve difficulty of scaling down by using general semiconductor techniques. In addition, corresponding silicon-based sealing cap structures are also demonstrated to replace traditional high cost metal lid. This silicon-based quartz resonator package is achieved by the Cu TSV interconnection, wafer thinning process, and sealing bonding techniques. With the combination of silicon-based design and quartz resonator, the advanced techniques of wafer level package to replace one chip sealing packaging are feasible and can offer low-cost packaging solution. The complete study of structure design, component characterization, and reliability evaluation for the innovated assembling design by using 3D integration scheme is presented to offer one potential solution for the next generation of quartz resonator components.Figure 1a shows the traditional quartz resonator component packaged in metal lid with ceramic-based interposer enclosures. In this paper, we demonstrate a novel silicon-based packaging approach by using 3D integration with general semiconductor techniques. This designed package is similar to conventional quartz resonator package but not metal lid with ceramic-based interposer assembling fabrication. Two novel silicon-based packages with quartz resonator using the concept of 3D integration are displayed in Figure 1b,c. The wafer level TSV interposer is implemented in both designs to replace traditional chip level ceramic-based interposer. The main difference of two silicon-based packages is about the silicon-based cap designs. Figure 1b is sealed with silicon cap through Cu/Sn eutectic bonding, and other is directly sealed by SU-8 polymer ring with polymer formed cavity in Figure 1c.

## Methods

### TSV interposer design

**Figure 2:**
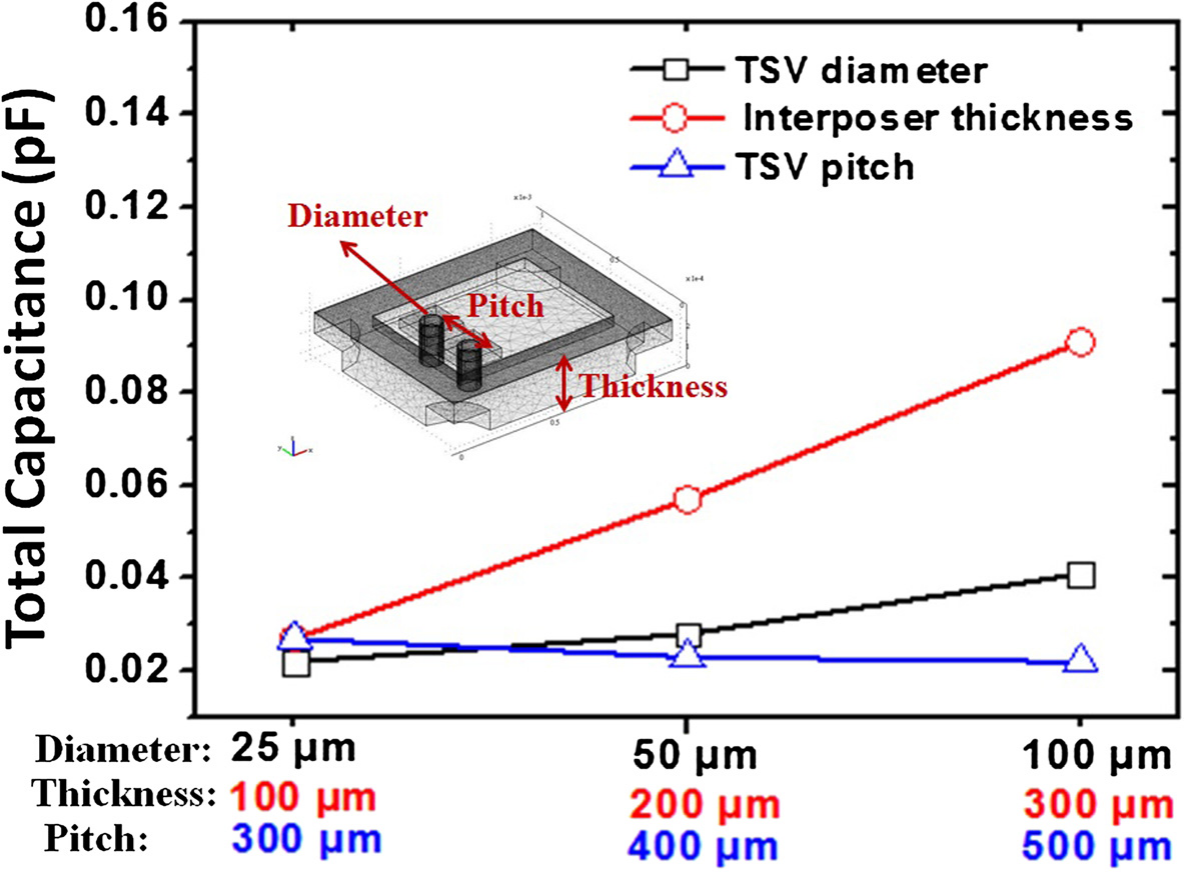
Simulation results of total capacitance characteristics for TSV diameter, pitch of TSV pairs, and thickness of Si-substrate.

Simulations about mechanical structure and electrical characteristics are performed to evaluate the design of TSV interposer. For the electrical characteristics, the total capacitances including self-capacitance and stray coupling capacitances are considered to evaluate the capacitance properties of TSV interposer by using Ansoft Q3D Extractor software (Ansys, Inc., Cecil Township, Pennsylvania, USA). The size of TSV interposer is 1.2 × 1.0 mm^2^ with different conditions of designed structural substrate, such as Cu TSV diameter, TSV pitch, and thickness of substrate are considered. Figure 2 shows the results of this structure using different dimensions of each component, where TSV diameters are 25, 50, and 100 μm under the fixed 100-μm interposer thickness and 300-μm TSV pitch; TSV pitches are 300, 400, and 500 μm under the fixed 25-μm TSV diameter and 300-μm TSV pitch; thicknesses of substrate are 100, 200, and 300 μm under the fixed 25-μm TSV diameter and 100-μm interposer thickness. The results show that interposer thickness is the main factor of the total capacitance in the whole TSV interposer design. However, the designs of TSV diameter and pitch have a tiny impact on total capacitance, compared to interposer thickness. Therefore, the capacitance of designed TSV interposer can be adjusted by changing the interposer thickness.

**Figure 3:**
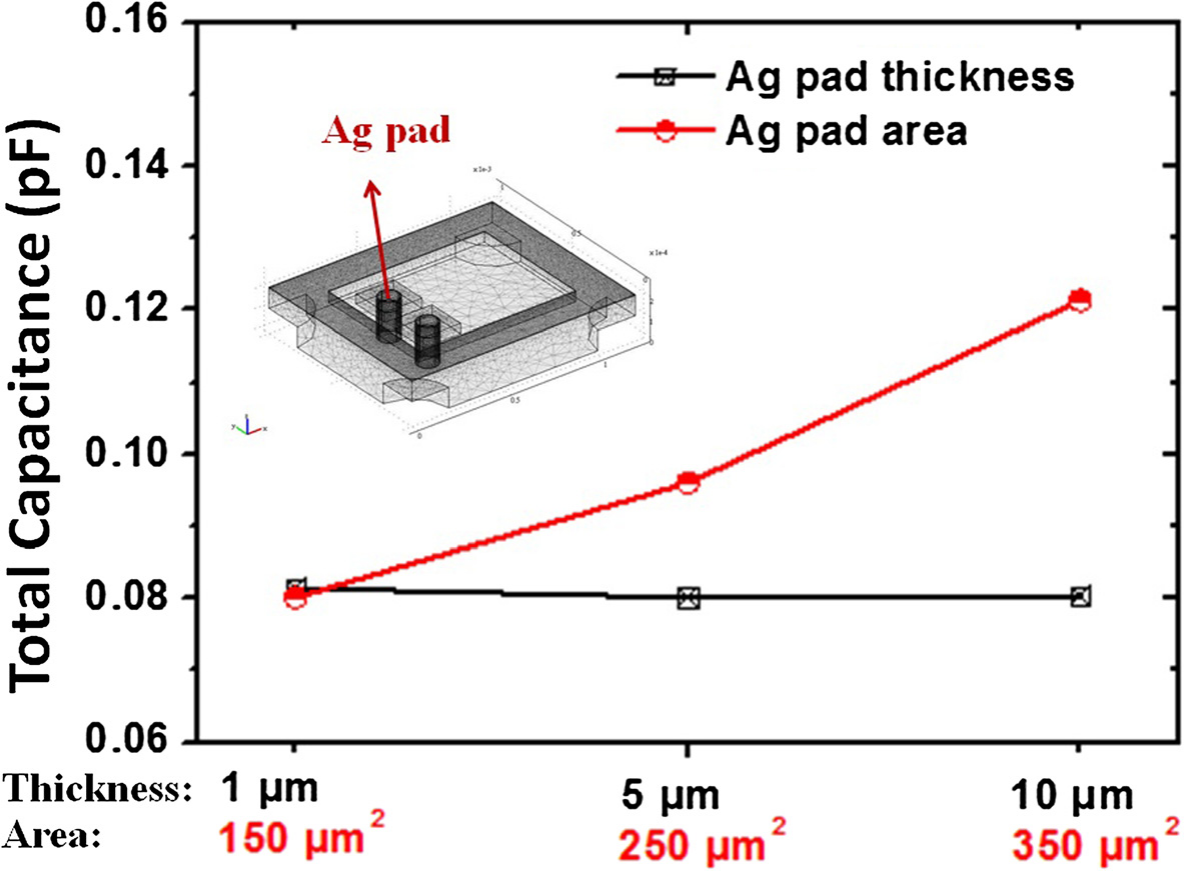
Simulation results of total capacitance characteristics for Ag pad thickness and area.

According to the above simulation results, the influence of total capacitance of Ag pad design is also evaluated under a different interposer size in a 1.2 × 1.0 mm^2^ substrate with 250-μm thickness and 100-μm diameter Cu TSVs. The Ag pad not only connects with Cu TSV but also provides the area to mount the quartz resonator with Ag conductive glue. Figure 3 shows the capacitance simulation results for Ag pad on TSV interposer based on altering pad area and thickness. Changing the Ag pad thickness has unapparent influence on total capacitance under the fixed 250-μm^2^ Ag pad area. However, Ag bump area has an obvious effect on total capacitance with fixed 1-μm Ag pad thickness. With capacitance simulation results, the whole capacitance of TSV interposer is adjustable in the manufacturing process.

**Figure 4:**
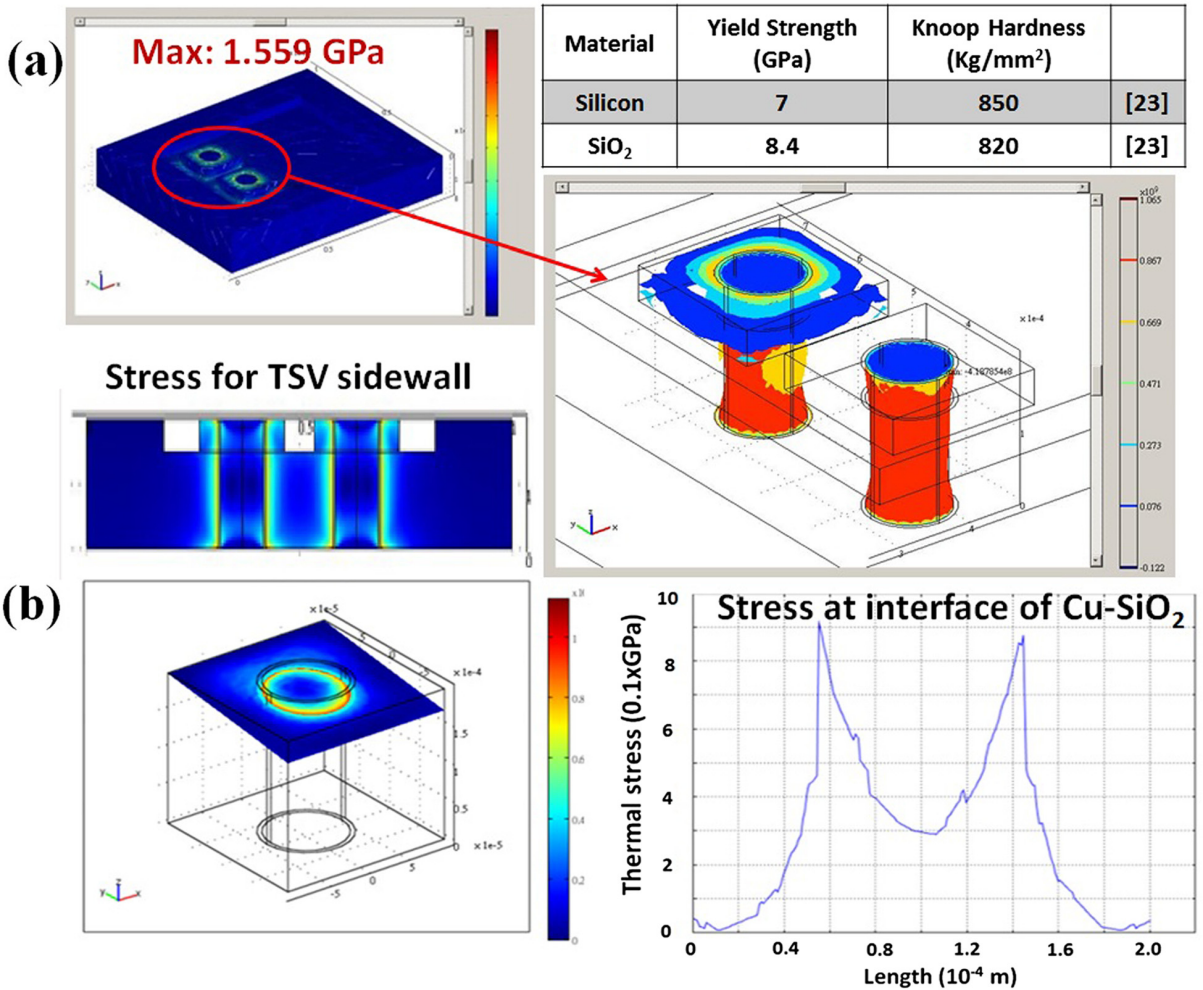
**Stress distribution for TSVs and the quantification of Cu TSV thermal stress results. (a)** Stress distribution for TSVs in the interposer based on thermal condition from room temperature to 700 K and **(b)** the quantification of Cu TSV thermal stress results.

On the other hand, the thermal stress simulation of designed TSV interposer is implemented by COMSOL Multiphysics software (COMSOL, Inc., Burlington, MA, USA) to evaluate thermal stress issues. In this case, thermal stress simulation contains thermal module and mechanical stress module simultaneously. The effect of a different interposer size in a 1.2 × 1.0 mm^2^ substrate with 250-μm thickness and 100-μm diameter Cu TSVs is evaluated by the thermal stress distribution at temperature condition from room temperature to 700 K. Because of the thermal budget of quartz resonator integration, the maximum temperature value is set up for 700 K. From the thermal stress simulation results in Figure 4a, it is clear that the main thermal stress occurs around Cu TSV profile of TSV interposer. In addition, with the quantification of thermal stress, the interface of Cu/SiO_2_ has the maximum thermal stress due to the coefficient of thermal expansion (CTE) mismatch in Figure 4b. In this study, although there is thermal stress around the sidewall of TSV, the value of maximum thermal stress is 1.559 GPa, which is still below yield strength of silicon for 7 GPa [23] (The yield strength of material can withstand without permanent deformation or fracture). For this reason, this interposer should be robust without cracking or deforming the TSV interposer under the thermal budget for 700 K. With the assistance of simulation results, the optimized TSV interposer can be achieved to integrate with quartz resonator.

### Component with TSV interposer

**Figure 5:**
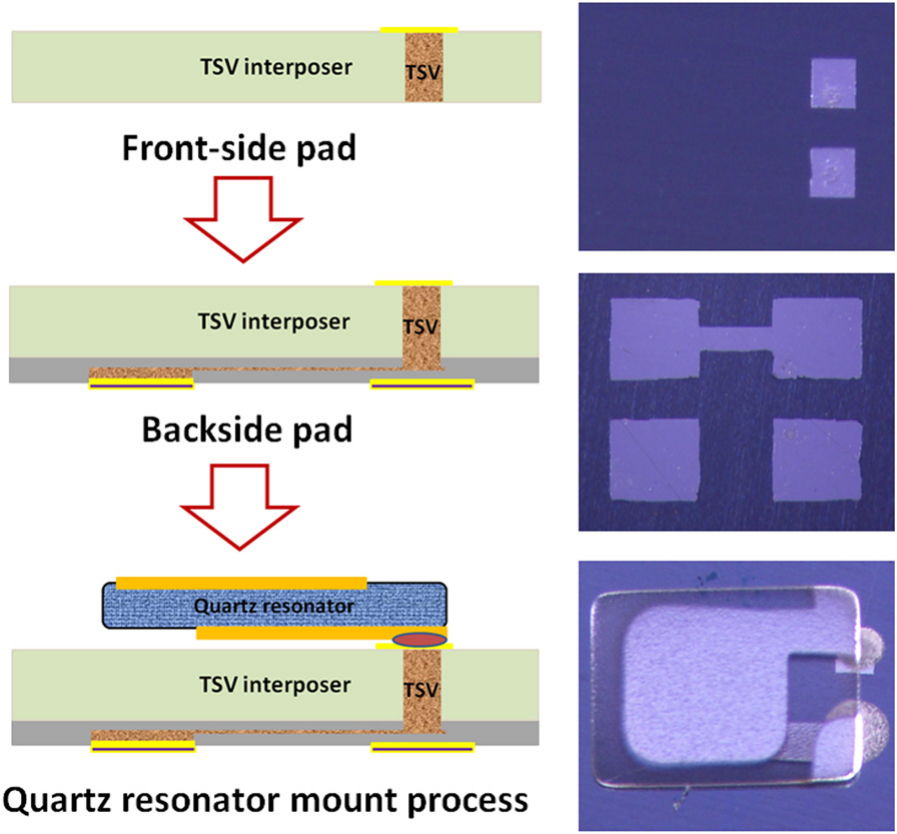
The integration for quartz resonator, TSV interposer, and Ag conductive glue.

After evaluating the designed interposer with simulation, the TSV interposer is demonstrated through the integration of Cu TSVs and wafer thinning process. The TSV interposer is fabricated with the design of 100-μm diameter and 250-μm depth Cu TSVs. To connect Cu TSVs, the redistribution metal layers are patterned on both the front-side for quartz resonator mount pad and on the backside for connection pad. Finally, the fabrication of TSV interposer is completed after patterning redistribution metal layers on both sides. The follow-up process is the heterogeneous integration between quartz resonator and TSV interposer. In order to integrate quartz resonator with designed interposer, the Ag conductive glue is applied on front-side Ag pad to attach quartz resonator, as shown in Figure 5. In order to ensure the quartz resonator fixed on front-side Ag pad of TSV interposer and avoid the collapsed Ag conductive glue occurring, a curing process is necessary.

Ag conductive glue is composed of Ag micro-ball with dense glue, which is not only for electrical connection between the quartz resonator and front-side Ag pad but also for providing the required space from silicon interposer for quartz resonator to operate mechanical vibration. In addition, the Ag conductive glue may release the thermal stress near front-side Ag pad surface and mechanical stress during quartz resonator vibration due to the flexible property. Therefore, this Ag conductive glue plays an important role in the heterogeneous integration between quartz resonator and TSV interposer. Overall, this novel design of quartz resonator with TSV interposer is fabricated successfully in wafer level process, and this scheme can provide the possibility to replace traditional one chip ceramic-based interposer.

### Thinned Si-cap and Si-polymer cap processing

**Figure 6:**
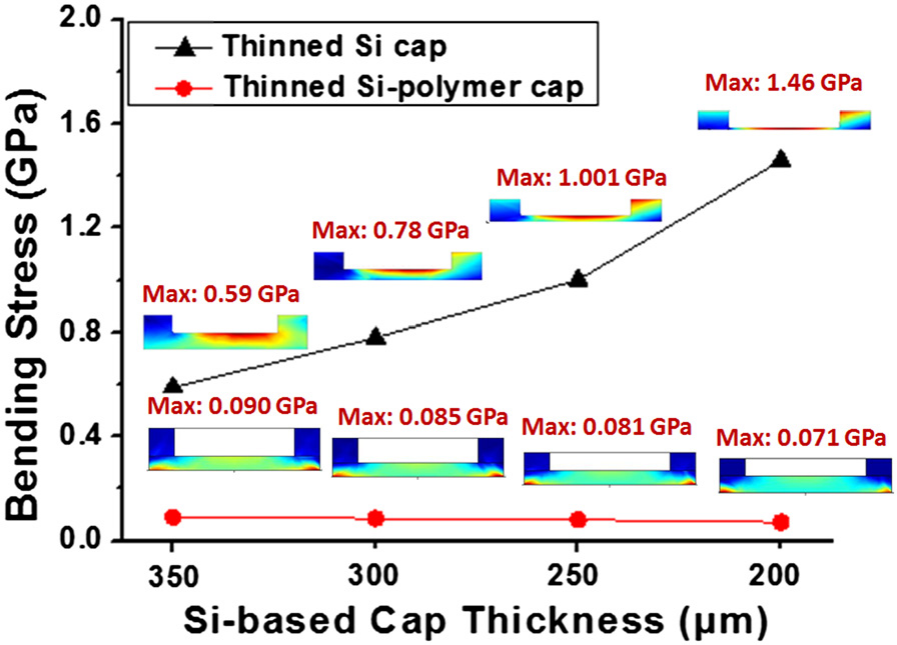
Bending simulation stress distribution of the thickness for thinned Si-cap and thinned Si-polymer cap.

For replacing the traditional metal lid of quartz resonator package, two types of silicon-based cap structure are developed with the same size of 1.2 × 1.0 mm^2^ cap and 0.84 × 0.64 mm^2^ cavity inside in general semiconductor techniques. Type 1 uses thinned Si-cap, which requires a protective cavity on silicon substrate by using deep reactive-ion etching (DRIE) method with Cu/Sn sealing ring around. Type 2 uses thinned Si-polymer cap, which uses SU-8 polymer ring as cap structure, providing sealing purpose by bonding without extra process. Before the fabrication of two cap structures, the mechanical reliability of two cap structures is considered for bending condition through COMSOL Multiphysics software (COMSOL, Inc., Burlington, MA, USA), as shown in Figure 6. In the beginning, the testing cap is placed on the middle of the bending broad with the fixed force for 2,450 Nt on broad. For the study of thinned Si-cap bending stress, the thinned Si-cap structure is evaluated by reducing the cap thickness from 400 to 200 μm with cavity depth of 180 μm. The maximum bending stress of thinned Si-cap always occurs at the four corners of inner cavity and decreases with increasing substrate thickness. Since the maximum bending stress of this structure is 1.559 GPa, which is still below the yield strength of silicon for 7 GPa [23], this thinned Si-cap design should be robust for the mechanical point of view.

**Figure 7:**
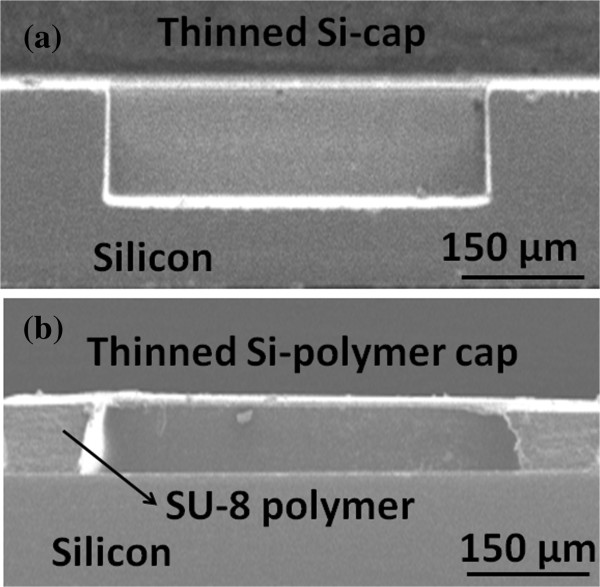
**The thinned Si-cap and thinned Si-polymer cap sealing structure. (a)** Thinned Si-cap. **(b)** Thinned Si-polymer cap.

In addition, the thinned Si-polymer cap structure with substrate thickness of 100 μm and SU-8 sealing ring thickness of 100 ~ 250 μm is also evaluated with the same bending condition. Obviously, the maximum bending stress of thinned Si-polymer cap is much smaller than thinned Si-cap cases. The results show the maximum bending stress of thinned Si-polymer cap always occurs at thinned Si substrate corners, but not at the interfaces of silicon and SU-8 polymer. Due to less mechanical stress compared to thinned Si-cap, this thinned Si-polymer cap is also robust as thinned Si-cap type without cracking or deforming substrate. Therefore, this bending simulation can provide a guideline for cap structure design among the two types. With the bending simulation results, the suitable cap structure can be achieved by replacing the metal lid of traditional quartz resonator.According to the results of bending simulation, the two types of cap structure are developed in each manufacturing process. For thinned Si-cap integration, silicon wafers with the thickness of 525 μm are thinned to 300-μm thick substrate in the beginning. Before thinned Si-cap wafer etching process, the Cu/Sn seal ring with the thickness of 4/2 μm is deposited on thinned Si-cap bonding areas for the next hermetic bonding purpose. Subsequently, the DRIE tool is used to form a depth of 150-μm cavities in wafer level using photoresist as etching mask. On the other hand, the fabrication of thinned Si-polymer cap is implemented and patterned on the 120-μm depth cavity structure through lithography process, as shown in Figure 7.

## Results and discussion

### Thermal stress issues

**Figure 8:**
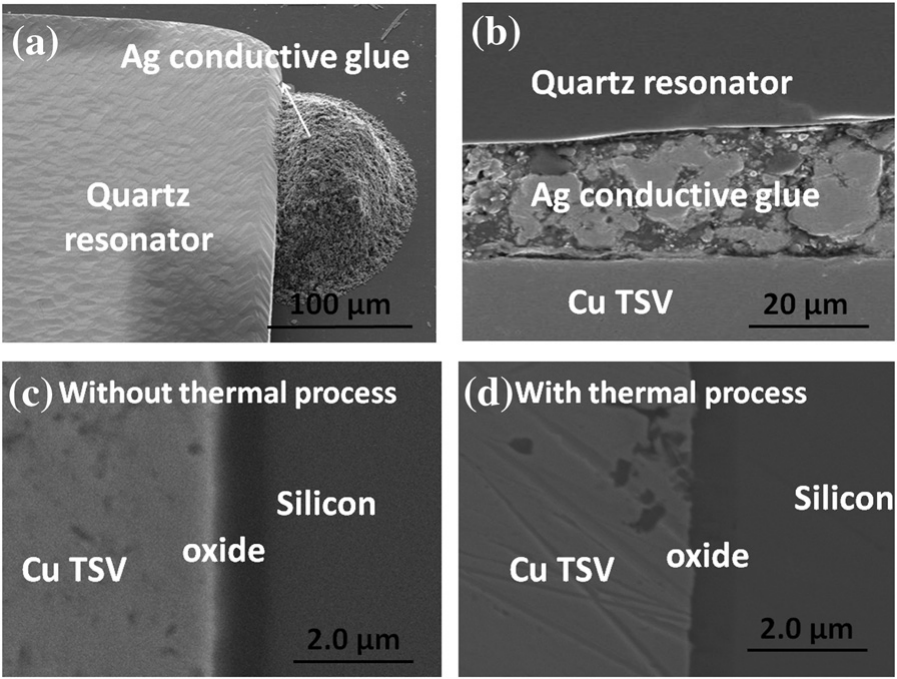
**The top view and cross-sectional SEM images of quartz resonator and Cu TSV profiles. (a,b)** The top view and cross-sectional SEM images of quartz resonator with TSV interposer after thermal reliability investigation and **(c,d)** the cross-sectional SEM image of Cu TSV profiles with and without thermal process.

Considering the thermal reliability of the integration of quartz resonator and this designed interposer, thermal process is implemented to inspect the quality of quartz resonator with TSV interposer. The thermal process is carried out with temperature condition from room temperature to 300°C for 24 h. Figure 8a,b shows the top view and cross-sectional scanning electron microscope (SEM) images of quartz resonator with TSV interposer after thermal reliability investigation. Without the deformations and cracks, the results indicate an excellent integrity for the integration of Cu TSV, Ag conductive glue and quartz resonator. In addition, the interfaces of Cu TSV/SiO_2_/Si layers are investigated with and without thermal process, as shown in Figure 8c,d. There are still no cracks or voids between the interfaces of TSV/SiO_2_/Si layers which indicate good quality of Cu TSV fabrication and are consistent with the above simulation results. Therefore, this thermal reliability results confirm a reliable and stable integration between quartz resonator and TSV interposer with thermal process investigation.

### Electrical characteristics

**Figure 9:**
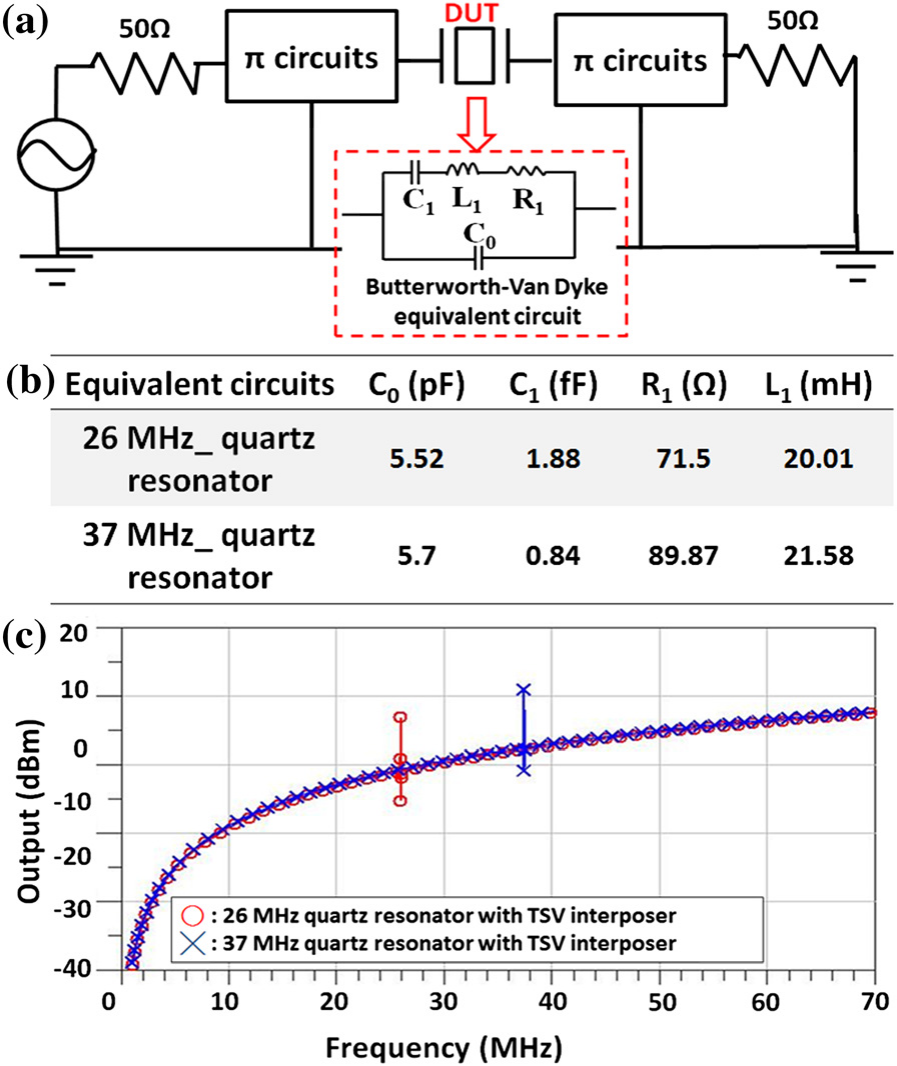
**Vector network analyzer with pi circuits, results of TSV interposer, and response output versus frequency with TSV interposer. (a)** Vector network analyzer with pi circuits, **(b)** measured results of TSV interposer with 26 and 37 MHz quartz resonator, and **(c)** the response output versus frequency of two standards of quartz resonator with TSV interposer.

Figure 9a shows the vector network analyzer with pi circuits for measurement of quartz resonator with TSV interposer. Since the vector network analyzer is used for conventional quartz resonator package, such as ceramic with metal lid type [2], it is used for investigating the electrical performances and frequency response of this novel integration of quartz resonator and TSV interposer. By using vector network analyzer, the equivalent circuit, Butterworth-Van Dyke, of silicon-based quartz resonator can be extracted. The frequency characteristics of two standard quartz resonators (26 and 37 MHz) integrated with TSV interposer are measured. Thus, Figure 9b can display the components of Butterworth-Van Dyke equivalent circuit, including C_1_, L_1_, R_1_, and C_0_, of two standard silicon-based quartz resonators.The frequency responses of each quartz resonator with TSV interposer are shown in Figure 9c. It is indicated that the specific oscillating frequency (approximately 26 and 37 MHz) can be outputted and employed to validate the feasibility in this research.

### Bonding reliability and pull test

**Figure 10:**
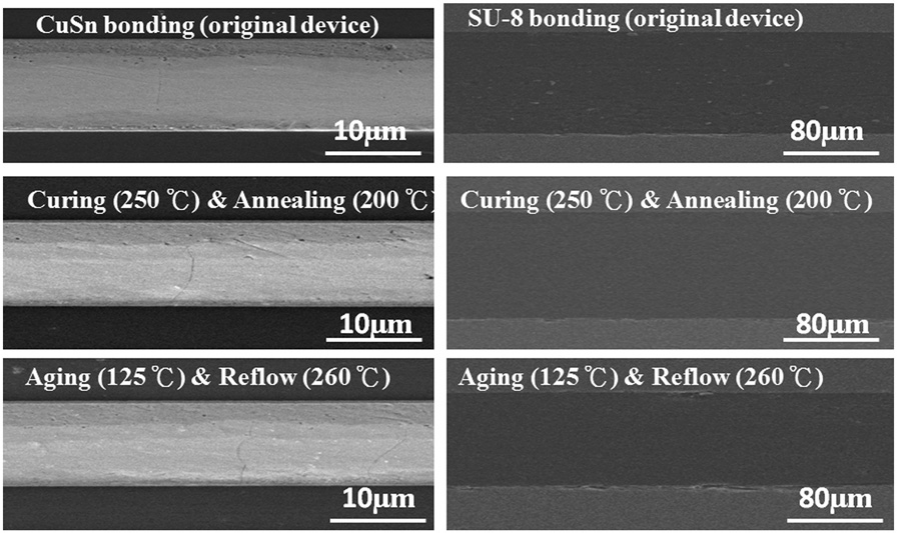
**The bonding interfaces of Cu/Sn layer and SU-8 polymer with thermal process investigation.** (left) The bonding interface of Cu/Sn layer with thermal process investigation and (right) the bonding interface of SU-8 polymer with thermal process investigation.

To study the further bonding reliability of two cap structures with the designed interposer, the thermal processes are implemented to examine the bonding interfaces of thinned Si-cap and thinned Si-polymer cap. The reliability tests of two type bonding interfaces are investigated in serial thermal process flow (annealing for 200°C, curing for 250°C, reflow for 260°C, and aging for 125°C) based on general quartz resonator thermal process. The two cap bonding interfaces are examined by the cross-sectional SEM images, as shown in Figure 10. It shows the excellent bonding integrity without voids or seams in two cap bonding interfaces for the follow-up thermal process. Based on the evidences of SEM profile, these two cap structures both show the robust bonded layer, which may be suitable cap sealing technologies with quartz blank for next generation of quartz resonator device.

In order to understand the mechanical strength of two cap bonding structures, the bonded cap structures (thinned Si-cap and thinned Si-polymer cap) with different temperature conditions are executed and then examined through pull test, as illustrated in Table 1. The pull test tool is set up with 15 × 15 mm bonded cap structures. The results of Si-cap show an excellent mechanical strength without de-bonds in the range of 250°C ~ 280°C but a failure at the location of original bonding interface after bonding 320°C and preliminary tests. It indicates that the Cu/Sn bonding structure remains intact under the bonding condition of 250°C ~ 280°C. On the other hand, the results of bonded thinned Si-polymer cap display the bonding interfaces which have acceptable enhancement under the bonding condition less than 250°C. However, when the bonding temperature is increased above the temperature of 300°C, the bonding mechanical strength decreases obviously. Due to the thermal budget limitation of quartz resonator limitation, the temperature of thermal/bonding process has to be as low as possible. These thinned Si-cap and thinned Si-polymer cap structures show the strong potential to replace the conventional metal lid. Furthermore, because of SU-8 polymer with lower temperature bonding condition, the thinned Si-polymer cap can achieve sealing process easily with a low temperature process, compared with traditional structures.

**Table 1:** Pull test results of bonded two cap structure with different temperature conditions

	**Bonding temperature (°C)**	**Pull force (kgf/cm**^**2**^**)**	**Failure location**	**Results**
Cu/Sn metal bonding	250	53.2	Fixture joint	Good for 250°C ~ 300°C
	280	47.7	Fixture joint	
	320	28.8	IMC/IMC	
	350	Failed	IMC/IMC	
SU-8 sealing bonding	200	30.57	Bonding joint	Good for low temperature but limiting for 250°C
	250	30.63	Bonding joint	
	300	11.4	Bonding joint	
	350	5.61	Bonding joint	

IMC, intermetallic compound.

Based on the advantages of cost efficient, easy to scale down, and wafer level sealing process, the thinned Si-cap and thinned Si-polymer cap structures have potential to offer feasible approach for the future advanced quartz resonator package.

## Conclusions

The optimum design, simulation, fabrication, and performances of wafer level packaging approach of quartz resonator based on TSV interposer with metal or polymer bonding are reported in this paper. This design is different from the conventional quartz resonator using ceramic-based package. This quartz resonator is designed with sealing bonding, wafer thinning, and Cu TSV interconnection. The silicon-based quartz resonator is able to meet the demands of small size and cost-effective technologies and also demonstrates the potential in advanced quartz resonator applications.

## Abbreviations

3D: three dimensional; Ag: silver; CTE: coefficient of thermal expansion; Cu: copper; DRIE: deep reactive ion etching; MEMS: micro electro mechanical systems; SEM: scanning electron microscope; Si: silicon; SiO_2_: silicon dioxide; Sn: tin; TSV: through-silicon via.

## Competing interests

The authors declare that they have no competing interests.

## Authors' contributions

J-YS wrote the manuscript and proceeded the research. J-YS and Y-CC integrated the quartz resonator with TSV interposer. C-HC, C-LL, and C-CC participated in the research concept. K-NC participated in the research concept, guided the research, and revised the manuscript. All authors read and approved the final manuscript.
